# (4-Cyano­phenolato)(subphthalocyaninato)boron^1^

**DOI:** 10.1107/S1600536811000869

**Published:** 2011-01-29

**Authors:** Andrew S. Paton, Alan J. Lough, Timothy P. Bender

**Affiliations:** a Department of Chemical Engineering & Applied Chemistry, University of Toronto, 200 College Street, Toronto, Ontario, Canada M5S 3E5; b Department of Chemistry, University of Toronto, 80 St. George Street, Toronto, Ontario, Canada M5S 3H6

## Abstract

The crystal structure of the title compound, C_31_H_16_BN_7_O, (CNPhO-BsubPc) is characterized by pairs of π–π stacking inter­actions between the concave faces of inversion-related BsubPc fragments with a centroid–centroid distance of 3.600 (1) Å. In addition, these pairs of mol­ecules are linked into chains along [101] through further weak π–π stacking inter­actions with a centroid–centroid distance of 3.8587 (9) Å. There are also weak C—H⋯π(arene) inter­actions within the chains.

## Related literature

For a general review of boronsubphthalocyanine compounds (BsubPcs), see: Claessens *et al.* (2002 ▶). For the synthesis of boronsubphthalocyanine and its derivatives, see: Zyskowski & Kennedy (2000 ▶); Claessens *et al.* (2003 ▶); Paton *et al.* (2011*b* ▶). For the application of BsubPcs in organic electronic devices, see: Morse *et al.* (2010 ▶) and references cited therein; Gommans *et al.* (2009) ▶. For related crystal structures of non-halogenated boronsubphthalocyanine derivatives, see: Potz *et al.* (2000 ▶); Paton *et al.* (2010 ▶, 2011*a* ▶,*b* ▶). For the treatment of disordered solvent mol­ecules, see: Athimoolam *et al.* (2005 ▶); Cox *et al.* (2003 ▶); Mohamed *et al.* (2003 ▶); Stähler *et al.* (2001 ▶).
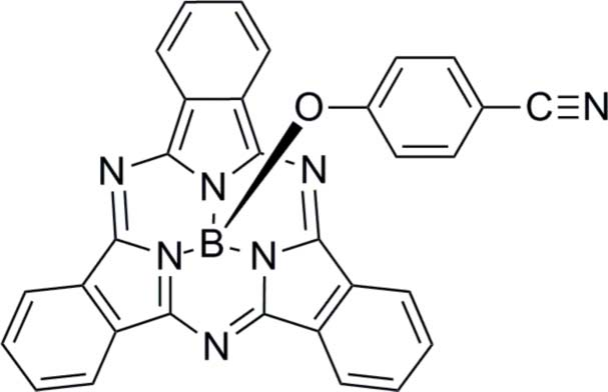


## Experimental

### 

#### Crystal data

C_31_H_16_BN_7_O
*M* *_r_* = 513.32
Monoclinic, 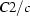

*a* = 16.2310 (3) Å
*b* = 27.5129 (7) Å
*c* = 13.4385 (2) Å
β = 119.4050 (12)°
*V* = 5228.00 (18) Å^3^
*Z* = 8
Mo *K*α radiation
μ = 0.08 mm^−1^
*T* = 150 K
0.40 × 0.30 × 0.12 mm

#### Data collection

Nonius KappaCCD diffractometer
Absorption correction: multi-scan (*SORTAV*; Blessing, 1995 ▶) *T*_min_ = 0.711, *T*_max_ = 0.994
21404 measured reflections
5914 independent reflections
4290 reflections with *I* > 2σ(*I*)
*R*_int_ = 0.087

#### Refinement

*R*[*F* ^2^ > 2σ(*F* ^2^)] = 0.049
*wR*(*F* ^2^) = 0.141
*S* = 1.06
5914 reflections
361 parameters
H-atom parameters constrained
Δρ_max_ = 0.23 e Å^−3^
Δρ_min_ = −0.26 e Å^−3^

### 

Data collection: *COLLECT* (Nonius, 2002 ▶); cell refinement: *DENZO-SMN* (Otwinowski & Minor, 1997 ▶); data reduction: *DENZO-SMN*; program(s) used to solve structure: *SIR92* (Altomare *et al.*, 1994 ▶); program(s) used to refine structure: *SHELXTL* (Sheldrick, 2008 ▶); molecular graphics: *PLATON* (Spek, 2009 ▶) and *Mercury* (Macrae *et al.*, 2008 ▶); software used to prepare material for publication: *SHELXTL*.

## Supplementary Material

Crystal structure: contains datablocks global, I. DOI: 10.1107/S1600536811000869/zl2323sup1.cif

Structure factors: contains datablocks I. DOI: 10.1107/S1600536811000869/zl2323Isup2.hkl

Additional supplementary materials:  crystallographic information; 3D view; checkCIF report

## Figures and Tables

**Table 1:** Hydrogen-bond geometry (Å, °) *Cg*1, *Cg*2 and *Cg*3 are the centroids of the C25–C30, N1/C1/C2/C7/C8 and N3/C9/C10/C15/C19 rings, respectively.

*D*—H⋯*A*	*D*—H	H⋯*A*	*D*⋯*A*	*D*—H⋯*A*
C3—H3*A*⋯*Cg*1^i^	0.95	2.70	3.499 (3)	143
C20—H20*A*⋯*Cg*2^ii^	0.95	2.59	3.254 (4)	127
C21—H21*A*⋯*Cg*3^ii^	0.95	2.70	3.238 (4)	116

Symmetry codes: (i) 
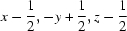
; (ii) 
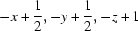
.

**Fig. 1.**
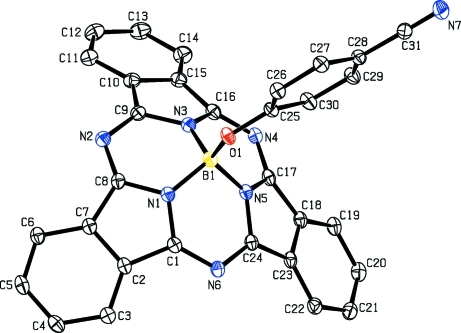
The labelled molecular structure of the title compound with displacement ellipsoids drawn at the 30% probability level. Hydrogen atoms were omitted for clarity.

**Fig. 2.**
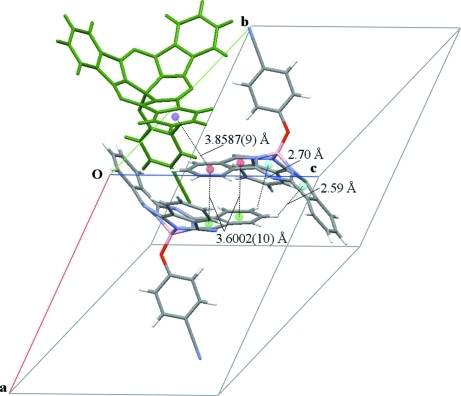
Part of the crystal structure of the title compound showing the π–π-stacking and C—H···π interactions as dotted lines.

**Fig. 3.**
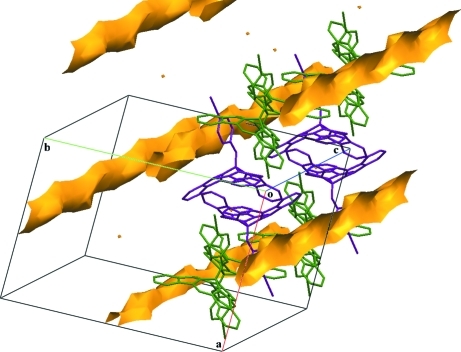
Part of the crystal structure of the title comound with the channel-like solvent voids (see experimental) CNPhO-BsubPc shown in orange.

## supplementary crystallographic information

### Comment

We report the crystal structure of 4-cyanophenoxy-boronsubphthalocyanine (CNPhO-BsubPc), which possesses an electron withdrawing group in the *para* position of the phenoxy molecular fragment. We have recently reported a study of the crystal structures of a series of *para*-substituted phenoxy-BsubPc wherein most of the substituents were electron-donating alkyl groups (Paton *et al.*, 2011*b*). Contained within that study was 4-fluorophenoxy-BsubPc (FPhO-BsubPc). While fluorine is moderately electron withdrawing we did not observe any difference in its crystal structure compared to the baseline phenoxy-BsubPc structure which contains pairs of molecules associated through π-stacking *via* the concave faces of two BsubPc molecules related by inversion centers. We have also have reported two BsubPc derivatives with electron-withdrawing groups in the *para* position, 4-acetylphenoxy-BsubPc (Paton *et al.*, 2010) and 4-nitrophenoxy-BsubPc (Paton *et al.*, 2011*a*). Both of these compounds are similar in structure to the FPhO-BsubPc, which typifies the substituted phenoxy-BsubPc packing motif.

The molecular structure of the title compound is shown in Fig. 1. The molecule shows the expected bowl shape of the BsubPc ligand. The B—O—C angle is 128.89 (11)°; however, both experimental and computational gas-phase values of B—O—C angles for phenoxy-derivatized BsubPc compounds have been shown to be significantly smaller, at 115.2 (2)° for the typical FPhO-BsubPc, and around 115° for the computationally determined gas-phase value (Paton *et al.*, 2011*b*). The torsion angle between the boron, oxygen, and the first two carbon atoms on the phenoxy substituent (B—O—C—C) is -1.9 (3)° while in FPhO-BsubPc it is -91.0 (2)°.

In the crystal structure, there are π···π interactions between the concave faces of pairs of molecules at a distance of 3.6002 (10) Å across an inversion centre (for rings N5/C17/C18/C23/C24 and C18—C23 related by 1/2-*x*, 1/2-*y*, 1-*z*). These types of π···π stacking interactions are common to other BsubPc derivatives mentioned above. In the crystal structure the title compound, additional weaker π···π-stacking interactions between inversion related pairs at a distance of 3.8587 (9) Å involving rings C18—C23 and C18—C23 related by 1-*x*, *y*, 3/2-*z*, form one-dimensional chains along [101].

Solvent voids are present in the structure (see experimental). The channel-like voids in which the solvent resides extend along the *c* axis (see Fig. 3) and are bordered by the convex-faces of the BsubPc fragment and the cyanophenoxy units. Diffusing heptane into a solution of the title compound in acetone, instead of heptane into a benzene solution as in the current experiment, gave the same crystal structure in terms of unit-cell parameters, cell volume, and solvent cavity volume.

### Experimental

Cl-BsubPc was synthesized by a procedure adapted from Zyskowski and Kennedy (2000). The title compound was synthesized using a method adapted from Claessens *et al.* (2003) and Paton *et al.* (2011*b*): 4-Cyanophenoxyboronsubphthalocyanine. Cl-BsubPc (0.510 g, 0.0012 mol) was mixed with 4-cyanophenol (0.714 g, 0.0060 mol) in toluene (10 ml) in a cylindrical vessel fitted with a reflux condenser and argon inlet. The mixture was stirred and heated at reflux under a constant pressure of argon for 17 h. Reaction was determined complete *via* HPLC by the absence of Cl-BsubPc. The solvent was evaporated under rotary evaporation. The crude product was purified on a Kauffman column using standard basic alumina (300 mesh) as the adsorbent and dichloromethane as the eluent. The product eluted from the Kauffman column while the excess phenol remained adsorbed. The dichloromethane was then removed under reduced pressure yielding a dark pink/magenta powder of the title compound (0.236 g, 40%).

### Refinement

All H atoms were placed in calculated positions and included in the refinment with C—H = 0.95Å and *U*_iso_(H) = 1.2*U*_eq_(C). During the refinement of the structure, electron density peaks (the largest being 2.38 e/Å^3^) were located that were believed to be highly disordered solvent molecules, possibly heptane and/or benzene. Attempts made to model the solvent molecule were not successful. The SQUEEZE option in *PLATON* (Spek, 2009) indicated there was a solvent cavity of volume 296 Å^3^ containing approximately 43 electrons per unit cell. We are not able to say with any certainty which of the two crystallization solvents used are present in the lattice. There was no observed streaking or satellite peaks on the exposed images to suggest that this might be a modulated srtructure. Therefore, in the final cycles of refinement, this contribution to the electron density was removed from the observed data. The density, the F(000) value, the molecular weight and the formula are given without taking into account the results obtained with the SQUEEZE option *PLATON* (Spek, 2009). Similar treatments of disordered solvent molecules were carried out by Stähler *et al.* (2001), Cox *et al.* (2003), Mohamed *et al.* (2003) and Athimoolam *et al.* (2005).

### Crystal data

**Table:**

C_31_H_16_BN_7_O	*F*(000) = 2112
*M**_r_* = 513.32	*D*_x_ = 1.304 Mg m^−^^3^
Monoclinic, *C*2/*c*	Mo *K*α radiation, λ = 0.71073 Å
Hall symbol: -C 2yc	Cell parameters from 21404 reflections
*a* = 16.2310 (3) Å	θ = 2.6–27.5°
*b* = 27.5129 (7) Å	µ = 0.08 mm^−^^1^
*c* = 13.4385 (2) Å	*T* = 150 K
β = 119.4050 (12)°	Plate, purple
*V* = 5228.00 (18) Å^3^	0.40 × 0.30 × 0.12 mm
*Z* = 8	

### Data collection

**Table:**

Nonius KappaCCD diffractometer	5914 independent reflections
Radiation source: fine-focus sealed tube	4290 reflections with *I* > 2σ(*I*)
graphite	*R*_int_ = 0.087
Detector resolution: 9 pixels mm^-1^	θ_max_ = 27.5°, θ_min_ = 2.7°
φ scans and ω scans with κ offsets	*h* = −21→18
Absorption correction: multi-scan (*SORTAV*; Blessing, 1995)	*k* = −35→35
*T*_min_ = 0.711, *T*_max_ = 0.994	*l* = −17→17
21404 measured reflections	

### Refinement

**Table:**

Refinement on *F*^2^	Primary atom site location: structure-invariant direct methods
Least-squares matrix: full	Secondary atom site location: difference Fourier map
*R*[*F*^2^ > 2σ(*F*^2^)] = 0.049	Hydrogen site location: inferred from neighbouring sites
*wR*(*F*^2^) = 0.141	H-atom parameters constrained
*S* = 1.06	*w* = 1/[σ^2^(*F*_o_^2^) + (0.0863*P*)^2^] where *P* = (*F*_o_^2^ + 2*F*_c_^2^)/3
5914 reflections	(Δ/σ)_max_ < 0.001
361 parameters	Δρ_max_ = 0.23 e Å^−^^3^
0 restraints	Δρ_min_ = −0.26 e Å^−^^3^

### Special details

**Table:**

Geometry. All e.s.d.'s (except the e.s.d. in the dihedral angle between two l.s. planes) are estimated using the full covariance matrix. The cell e.s.d.'s are taken into account individually in the estimation of e.s.d.'s in distances, angles and torsion angles; correlations between e.s.d.'s in cell parameters are only used when they are defined by crystal symmetry. An approximate (isotropic) treatment of cell e.s.d.'s is used for estimating e.s.d.'s involving l.s. planes.
Refinement. Refinement of *F*^2^ against ALL reflections. The weighted *R*-factor *wR* and goodness of fit *S* are based on *F*^2^, conventional *R*-factors *R* are based on *F*, with *F* set to zero for negative *F*^2^. The threshold expression of *F*^2^ > σ(*F*^2^) is used only for calculating *R*-factors(gt) *etc*. and is not relevant to the choice of reflections for refinement. *R*-factors based on *F*^2^ are statistically about twice as large as those based on *F*, and *R*- factors based on ALL data will be even larger.

### Fractional atomic coordinates and isotropic or equivalent isotropic displacement parameters (Å^2^)

**Table:**

	*x*	*y*	*z*	*U*_iso_*/*U*_eq_	
O1	0.45381 (6)	0.13599 (4)	0.37150 (8)	0.0282 (3)	
N1	0.29842 (7)	0.16745 (5)	0.25275 (9)	0.0239 (3)	
N2	0.20587 (8)	0.09502 (5)	0.20043 (10)	0.0279 (3)	
N3	0.31564 (8)	0.11128 (5)	0.39600 (10)	0.0250 (3)	
N4	0.34779 (8)	0.14317 (5)	0.57623 (10)	0.0269 (3)	
N5	0.37213 (7)	0.19169 (5)	0.44565 (9)	0.0234 (3)	
N6	0.32295 (8)	0.25170 (5)	0.30000 (10)	0.0256 (3)	
N7	0.89695 (9)	0.09629 (6)	0.79845 (12)	0.0423 (4)	
C1	0.28553 (9)	0.21531 (6)	0.22298 (12)	0.0244 (3)	
C2	0.20815 (9)	0.21704 (6)	0.10579 (11)	0.0246 (3)	
C3	0.16247 (10)	0.25605 (6)	0.03201 (12)	0.0276 (3)	
H3A	0.1862	0.2883	0.0515	0.033*	
C4	0.08163 (11)	0.24607 (6)	−0.07024 (12)	0.0319 (4)	
H4A	0.0503	0.2718	−0.1225	0.038*	
C5	0.04511 (10)	0.19906 (6)	−0.09831 (12)	0.0324 (4)	
H5A	−0.0114	0.1937	−0.1685	0.039*	
C6	0.08894 (10)	0.16012 (6)	−0.02685 (12)	0.0285 (3)	
H6A	0.0637	0.1282	−0.0467	0.034*	
C7	0.17179 (9)	0.16932 (6)	0.07568 (11)	0.0249 (3)	
C8	0.22770 (9)	0.13839 (6)	0.17416 (11)	0.0245 (3)	
C9	0.24642 (10)	0.08345 (6)	0.31192 (12)	0.0266 (3)	
C10	0.21193 (10)	0.05125 (6)	0.36928 (12)	0.0295 (3)	
C11	0.14170 (11)	0.01560 (6)	0.32724 (14)	0.0343 (4)	
H11A	0.1111	0.0063	0.2491	0.041*	
C12	0.11801 (12)	−0.00584 (6)	0.40292 (15)	0.0401 (4)	
H12A	0.0717	−0.0308	0.3765	0.048*	
C13	0.16061 (13)	0.00841 (7)	0.51697 (15)	0.0411 (4)	
H13A	0.1427	−0.0070	0.5667	0.049*	
C14	0.22856 (12)	0.04466 (6)	0.55934 (14)	0.0371 (4)	
H14A	0.2566	0.0546	0.6369	0.044*	
C15	0.25479 (10)	0.06624 (6)	0.48518 (12)	0.0294 (4)	
C16	0.31566 (10)	0.10753 (6)	0.49800 (12)	0.0272 (3)	
C17	0.36934 (9)	0.18587 (6)	0.54540 (11)	0.0250 (3)	
C18	0.37682 (9)	0.23457 (6)	0.59076 (11)	0.0256 (3)	
C19	0.38193 (9)	0.25146 (6)	0.69168 (12)	0.0290 (4)	
H19A	0.3810	0.2296	0.7459	0.035*	
C20	0.38842 (10)	0.30100 (6)	0.70979 (13)	0.0320 (4)	
H20A	0.3938	0.3133	0.7788	0.038*	
C21	0.38732 (10)	0.33359 (6)	0.62963 (13)	0.0333 (4)	
H21A	0.3933	0.3674	0.6459	0.040*	
C22	0.37760 (9)	0.31749 (6)	0.52658 (13)	0.0309 (4)	
H22A	0.3741	0.3398	0.4707	0.037*	
C23	0.37317 (9)	0.26767 (6)	0.50768 (11)	0.0257 (3)	
C24	0.36076 (9)	0.23903 (6)	0.41071 (12)	0.0244 (3)	
C25	0.54079 (9)	0.12725 (6)	0.46227 (12)	0.0252 (3)	
C26	0.61048 (10)	0.11309 (6)	0.43571 (12)	0.0280 (3)	
H26A	0.5952	0.1096	0.3582	0.034*	
C27	0.70191 (10)	0.10419 (6)	0.52242 (12)	0.0286 (3)	
H27A	0.7487	0.0937	0.5042	0.034*	
C28	0.72545 (10)	0.11047 (6)	0.63614 (12)	0.0275 (3)	
C29	0.65582 (11)	0.12432 (6)	0.66259 (13)	0.0316 (4)	
H29A	0.6715	0.1285	0.7401	0.038*	
C30	0.56400 (10)	0.13197 (6)	0.57646 (12)	0.0306 (4)	
H30A	0.5165	0.1405	0.5951	0.037*	
C31	0.82104 (11)	0.10284 (6)	0.72633 (13)	0.0308 (4)	
B1	0.36726 (11)	0.15027 (7)	0.37081 (13)	0.0256 (4)	

### Atomic displacement parameters (Å^2^)

**Table:**

	*U*^11^	*U*^22^	*U*^33^	*U*^12^	*U*^13^	*U*^23^
O1	0.0217 (5)	0.0396 (7)	0.0226 (5)	0.0049 (4)	0.0102 (4)	0.0000 (4)
N1	0.0231 (6)	0.0279 (8)	0.0215 (6)	0.0008 (5)	0.0115 (5)	−0.0012 (5)
N2	0.0302 (6)	0.0288 (8)	0.0261 (6)	0.0013 (5)	0.0150 (5)	−0.0032 (5)
N3	0.0261 (6)	0.0270 (7)	0.0226 (6)	0.0028 (5)	0.0124 (5)	−0.0001 (5)
N4	0.0241 (6)	0.0323 (8)	0.0236 (6)	0.0010 (5)	0.0111 (5)	−0.0002 (5)
N5	0.0199 (5)	0.0293 (8)	0.0206 (6)	0.0001 (5)	0.0096 (5)	−0.0011 (5)
N6	0.0215 (6)	0.0315 (8)	0.0245 (6)	−0.0016 (5)	0.0118 (5)	−0.0011 (5)
N7	0.0293 (7)	0.0455 (10)	0.0408 (8)	0.0011 (6)	0.0085 (7)	−0.0024 (7)
C1	0.0202 (7)	0.0311 (9)	0.0247 (7)	0.0004 (6)	0.0132 (6)	0.0009 (6)
C2	0.0210 (7)	0.0342 (9)	0.0207 (7)	0.0003 (6)	0.0118 (6)	−0.0003 (6)
C3	0.0263 (7)	0.0321 (9)	0.0267 (7)	0.0005 (6)	0.0148 (6)	0.0001 (6)
C4	0.0284 (8)	0.0426 (11)	0.0246 (7)	0.0067 (7)	0.0130 (6)	0.0051 (7)
C5	0.0241 (7)	0.0480 (11)	0.0218 (7)	0.0025 (7)	0.0086 (6)	−0.0022 (7)
C6	0.0259 (7)	0.0362 (10)	0.0240 (7)	0.0006 (6)	0.0126 (6)	−0.0041 (6)
C7	0.0238 (7)	0.0320 (9)	0.0223 (7)	0.0020 (6)	0.0139 (6)	−0.0027 (6)
C8	0.0235 (7)	0.0296 (9)	0.0225 (7)	0.0006 (6)	0.0130 (6)	−0.0045 (6)
C9	0.0290 (7)	0.0254 (9)	0.0270 (7)	0.0025 (6)	0.0151 (6)	−0.0027 (6)
C10	0.0334 (8)	0.0272 (9)	0.0311 (8)	0.0034 (6)	0.0183 (7)	−0.0004 (6)
C11	0.0360 (8)	0.0312 (10)	0.0364 (9)	0.0013 (7)	0.0183 (7)	−0.0005 (7)
C12	0.0448 (10)	0.0310 (10)	0.0494 (10)	−0.0049 (7)	0.0270 (8)	0.0001 (8)
C13	0.0520 (10)	0.0345 (11)	0.0458 (10)	−0.0010 (8)	0.0310 (9)	0.0073 (8)
C14	0.0452 (9)	0.0367 (11)	0.0341 (8)	−0.0003 (8)	0.0231 (8)	0.0027 (7)
C15	0.0309 (8)	0.0292 (9)	0.0297 (8)	0.0038 (6)	0.0160 (7)	0.0020 (6)
C16	0.0264 (7)	0.0329 (9)	0.0229 (7)	0.0037 (6)	0.0127 (6)	0.0013 (6)
C17	0.0183 (6)	0.0356 (10)	0.0204 (7)	0.0022 (6)	0.0089 (6)	−0.0005 (6)
C18	0.0168 (6)	0.0345 (9)	0.0246 (7)	0.0004 (6)	0.0096 (6)	−0.0032 (6)
C19	0.0198 (7)	0.0419 (10)	0.0267 (8)	0.0002 (6)	0.0124 (6)	−0.0041 (7)
C20	0.0217 (7)	0.0444 (11)	0.0312 (8)	0.0017 (6)	0.0141 (6)	−0.0102 (7)
C21	0.0233 (7)	0.0345 (10)	0.0379 (9)	0.0020 (6)	0.0117 (7)	−0.0099 (7)
C22	0.0227 (7)	0.0337 (10)	0.0321 (8)	0.0013 (6)	0.0103 (6)	−0.0024 (7)
C23	0.0173 (6)	0.0329 (9)	0.0246 (7)	−0.0006 (6)	0.0084 (6)	−0.0041 (6)
C24	0.0173 (6)	0.0306 (9)	0.0248 (7)	−0.0006 (6)	0.0100 (6)	−0.0011 (6)
C25	0.0218 (7)	0.0288 (9)	0.0246 (7)	0.0022 (6)	0.0111 (6)	0.0031 (6)
C26	0.0278 (7)	0.0336 (10)	0.0255 (7)	0.0010 (6)	0.0152 (6)	0.0004 (6)
C27	0.0242 (7)	0.0338 (10)	0.0311 (8)	0.0006 (6)	0.0160 (6)	0.0013 (6)
C28	0.0237 (7)	0.0283 (9)	0.0277 (8)	0.0012 (6)	0.0104 (6)	0.0015 (6)
C29	0.0319 (8)	0.0380 (10)	0.0238 (8)	0.0063 (7)	0.0128 (7)	0.0005 (6)
C30	0.0269 (7)	0.0419 (10)	0.0253 (8)	0.0082 (7)	0.0146 (6)	0.0024 (7)
C31	0.0292 (8)	0.0309 (10)	0.0299 (8)	−0.0009 (6)	0.0127 (7)	−0.0023 (7)
B1	0.0237 (8)	0.0300 (10)	0.0233 (8)	0.0033 (7)	0.0117 (7)	−0.0013 (7)

### Geometric parameters (Å, °)

**Table:**

O1—C25	1.3593 (16)	C11—C12	1.384 (2)
O1—B1	1.4544 (18)	C11—H11A	0.9500
N1—C1	1.362 (2)	C12—C13	1.393 (2)
N1—C8	1.3718 (18)	C12—H12A	0.9500
N1—B1	1.4995 (19)	C13—C14	1.385 (2)
N2—C8	1.340 (2)	C13—H13A	0.9500
N2—C9	1.3458 (18)	C14—C15	1.394 (2)
N3—C9	1.3706 (18)	C14—H14A	0.9500
N3—C16	1.3744 (18)	C15—C16	1.459 (2)
N3—B1	1.499 (2)	C17—C18	1.452 (2)
N4—C16	1.342 (2)	C18—C19	1.397 (2)
N4—C17	1.348 (2)	C18—C23	1.419 (2)
N5—C24	1.3658 (19)	C19—C20	1.379 (2)
N5—C17	1.3726 (18)	C19—H19A	0.9500
N5—B1	1.496 (2)	C20—C21	1.395 (2)
N6—C24	1.3473 (18)	C20—H20A	0.9500
N6—C1	1.3510 (19)	C21—C22	1.387 (2)
N7—C31	1.1476 (19)	C21—H21A	0.9500
C1—C2	1.4553 (18)	C22—C23	1.389 (2)
C2—C3	1.401 (2)	C22—H22A	0.9500
C2—C7	1.415 (2)	C23—C24	1.450 (2)
C3—C4	1.384 (2)	C25—C30	1.395 (2)
C3—H3A	0.9500	C25—C26	1.398 (2)
C4—C5	1.395 (2)	C26—C27	1.386 (2)
C4—H4A	0.9500	C26—H26A	0.9500
C5—C6	1.380 (2)	C27—C28	1.392 (2)
C5—H5A	0.9500	C27—H27A	0.9500
C6—C7	1.3969 (19)	C28—C29	1.394 (2)
C6—H6A	0.9500	C28—C31	1.440 (2)
C7—C8	1.456 (2)	C29—C30	1.382 (2)
C9—C10	1.455 (2)	C29—H29A	0.9500
C10—C11	1.396 (2)	C30—H30A	0.9500
C10—C15	1.420 (2)		
			
C25—O1—B1	128.89 (11)	C14—C15—C10	120.23 (14)
C1—N1—C8	112.80 (11)	C14—C15—C16	132.36 (15)
C1—N1—B1	122.86 (12)	C10—C15—C16	107.11 (13)
C8—N1—B1	122.55 (13)	N4—C16—N3	122.43 (14)
C8—N2—C9	117.12 (12)	N4—C16—C15	129.92 (14)
C9—N3—C16	112.27 (12)	N3—C16—C15	105.78 (13)
C9—N3—B1	122.45 (12)	N4—C17—N5	122.68 (13)
C16—N3—B1	123.54 (12)	N4—C17—C18	130.99 (13)
C16—N4—C17	117.12 (13)	N5—C17—C18	105.44 (13)
C24—N5—C17	112.54 (12)	C19—C18—C23	120.65 (15)
C24—N5—B1	122.79 (12)	C19—C18—C17	131.87 (15)
C17—N5—B1	123.46 (13)	C23—C18—C17	107.41 (12)
C24—N6—C1	116.35 (13)	C20—C19—C18	117.58 (15)
N6—C1—N1	123.02 (12)	C20—C19—H19A	121.2
N6—C1—C2	129.00 (14)	C18—C19—H19A	121.2
N1—C1—C2	106.01 (12)	C19—C20—C21	121.86 (14)
C3—C2—C7	120.47 (13)	C19—C20—H20A	119.1
C3—C2—C1	131.80 (14)	C21—C20—H20A	119.1
C7—C2—C1	107.24 (13)	C22—C21—C20	121.19 (16)
C4—C3—C2	117.71 (15)	C22—C21—H21A	119.4
C4—C3—H3A	121.1	C20—C21—H21A	119.4
C2—C3—H3A	121.1	C21—C22—C23	117.89 (15)
C3—C4—C5	121.48 (15)	C21—C22—H22A	121.1
C3—C4—H4A	119.3	C23—C22—H22A	121.1
C5—C4—H4A	119.3	C22—C23—C18	120.72 (13)
C6—C5—C4	121.73 (14)	C22—C23—C24	132.25 (14)
C6—C5—H5A	119.1	C18—C23—C24	106.98 (14)
C4—C5—H5A	119.1	N6—C24—N5	122.53 (13)
C5—C6—C7	117.60 (15)	N6—C24—C23	129.70 (14)
C5—C6—H6A	121.2	N5—C24—C23	105.88 (12)
C7—C6—H6A	121.2	O1—C25—C30	124.84 (13)
C6—C7—C2	120.97 (13)	O1—C25—C26	115.73 (12)
C6—C7—C8	131.24 (15)	C30—C25—C26	119.42 (13)
C2—C7—C8	107.23 (12)	C27—C26—C25	120.06 (13)
N2—C8—N1	123.06 (13)	C27—C26—H26A	120.0
N2—C8—C7	129.22 (13)	C25—C26—H26A	120.0
N1—C8—C7	105.76 (13)	C26—C27—C28	120.29 (14)
N2—C9—N3	122.72 (14)	C26—C27—H27A	119.9
N2—C9—C10	128.97 (13)	C28—C27—H27A	119.9
N3—C9—C10	106.04 (12)	C27—C28—C29	119.60 (13)
C11—C10—C15	120.73 (14)	C27—C28—C31	120.48 (13)
C11—C10—C9	131.58 (14)	C29—C28—C31	119.91 (14)
C15—C10—C9	107.20 (13)	C30—C29—C28	120.25 (14)
C12—C11—C10	117.94 (15)	C30—C29—H29A	119.9
C12—C11—H11A	121.0	C28—C29—H29A	119.9
C10—C11—H11A	121.0	C29—C30—C25	120.32 (14)
C11—C12—C13	121.46 (16)	C29—C30—H30A	119.8
C11—C12—H12A	119.3	C25—C30—H30A	119.8
C13—C12—H12A	119.3	N7—C31—C28	179.29 (19)
C14—C13—C12	121.33 (16)	O1—B1—N5	117.99 (12)
C14—C13—H13A	119.3	O1—B1—N3	116.79 (13)
C12—C13—H13A	119.3	N5—B1—N3	104.14 (12)
C13—C14—C15	118.27 (15)	O1—B1—N1	107.91 (12)
C13—C14—H14A	120.9	N5—B1—N1	103.72 (12)
C15—C14—H14A	120.9	N3—B1—N1	104.83 (12)

### Hydrogen-bond geometry (Å, °)

**Table:**

Cg1, Cg2 and Cg3 are the centroids of the C25–C30, N1/C1/C2/C7/C8 and N3/C9/C10/C15/C19 rings, respectively.

**Table:**

*D*—H···*A*	*D*—H	H···*A*	*D*···*A*	*D*—H···*A*
C3—H3A···Cg1^i^	0.95	2.70	3.499 (3)	143
C20—H20A···Cg2^ii^	0.95	2.59	3.254 (4)	127
C21—H21A···Cg3^ii^	0.95	2.70	3.238 (4)	116

Symmetry codes: (i) *x*−1/2, −*y*+1/2, *z*−1/2; (ii) −*x*+1/2, −*y*+1/2, −*z*+1.
